# Cooperative light scattering from helical-phase-imprinted atomic rings

**DOI:** 10.1038/s41598-018-27888-y

**Published:** 2018-06-22

**Authors:** H. H. Jen, M.-S. Chang, Y.-C. Chen

**Affiliations:** 1 Institute of Physics, Academia Sinica, Taipei, 11529 Taiwan; 2 Institute of Atomic and Molecular Sciences, Academia Sinica, Taipei, 10617 Taiwan

## Abstract

We theoretically investigate the light scattering of super- and subradiant states of an atomic ring prepared by single excitation with a photon which carries an orbital angular momentum (OAM). For excitations with linear polarizations, the helical phase imprinted (HPI) atomic ring presents a discrete *C*_4_ rotational symmetry when number of atoms *N* = 4*n* with integers *n*, while for circular polarizations with arbitrary *N*, the continuous and *C*_*N*_ symmetries emerge for the super- and subradiant modes, respectively. The HPI superradiant modes predominantly scatter photons in the forward-backward direction, and the forward scattering can be further enhanced as atomic rings are stacked along the excitation direction. The HPI subradiant modes then preferentially scatter photons in the transversal directions, and when rings are stacked concentrically and on a plane, crossover from sub- to superradiance is observed which leads to splitting and localization of the far-field scattering patterns in the polar angle. The HPI super- and subradiant states are thus detectable through measuring the far-field radiation patterns, which further allow quantum storage and detection of a single photon with an OAM.

## Introduction

Controlled strong light-matter interactions in quantum optical systems for efficient generation, storage, and manipulation of quantum correlations^1^ is essential for establishing robust long-distance quantum entanglement for quantum communication^2–4^ and quantum network^5^. This has also spurred the development of quantum memory and quantum repeater in recent years^6^, which stores and relays entanglement. Quantum correlation in an atomic system is often arisen through spontaneous emissions, in which the atomic ensemble collectively emits a photon following an atomic excitation. This serves an elementary mechanism to entangle the atomic states with discrete states of light, such as polarizations^7–9^. This bipartite entanglement can also be generated in the biphoton states in spatial modes, or energy-time^10^, using means including parametric down conversion from nonlinear crystals^11,12^, cascade emissions in atoms^13–16^, or correlated light beams from a rare-earth ion-doped crystal^17^.

To increase the efficiency of light-matter interaction, directional spontaneous emissions are enhanced in optically thick atomic ensembles^18–20^ through superradiance^21,22^, utilizing the resonant and pairwise dipole-dipole interactions^23,24^ among the atoms in the dissipation process. This collective light-matter interaction also results in a frequency shift^25–33^ and is responsible for subradiant radiations^34^ as an afterglow of superradiance^35^. In the context of quantum memory, subradiant states are candidate systems for storing photons and can be actively prepared in a dense medium^36–41^, through selective radiance by controlling the density and/or geometry of an array of atoms or metamolecules^42–44^, collective antiresonances from the subradiant arrays in a cavity^45^, or through creating quantum optical analogs of topological states in two-dimensional atomic arrays^46^. The light scattering from the subradiant states is also under active investigations recently^20,34^, but a systematic and detailed study on the subradiant modes, which is essential for efficient assess to those modes, is still lacking.

The rapid development on precisely positioning single atoms utilizing photonic crystal waveguide^47^, optical microtraps^48,49^, or creating an array of artificial atoms in solid-state nanophotonic platforms^50^ has further enabled fabrication of atomic ensembles with arbitrary spatial distributions beyond the diffraction limit of the excitation field, thus offers new opportunities to explore super- and subradiant modes, and opens up a new avenue for tailoring and modifying the quantum states of light and matter. In this paper, we propose to prepare the phase-imprinted single-photon subradiant states in the stacked ring arrays of atoms, in which light with orbital angular momentum (OAM)^51–55^ interacts with the atoms, and the helical-phase-imprinted (HPI) subradiant states can be prepared upon absorption. The HPI subradiant states thus serves as good candidates for storing light quanta with OAM^56–58^. We investigate the light scattering out of these subradiant states for the cases of a few atoms, a single ring, and stacked rings. We also study the effect of uniform and spatially-dependent light polarizations on the scattering patterns.

## Helical-Phase-Imprinted Subradiant States

When a near-resonant single photon is absorbed by an ensemble of *N* two-level atoms, a symmetric state is formed,

1 $$|{{\rm{\Phi }}}_{N}\rangle =\frac{1}{\sqrt{N}}\,\sum _{\mu =1}^{N}\,{e}^{i{{\bf{k}}}_{L}\cdot {{\bf{r}}}_{\mu }}|e{\rangle }_{\mu }|g{\rangle }^{\otimes (N-1)},$$

where |*g*〉 and |*e*〉 label the ground and excited states of a two-level atom, and **k**_*L*_ is the wave vector of the plane-wave excitation field. Here, each of the two-level atoms can be promoted to the excited state with an equal probability *N*^−1^ and acquires a position-dependent traveling phase ${e}^{i{{\bf{k}}}_{L}\cdot {{\bf{r}}}_{\mu }}$. This symmetric state can be superradiant when the inter-atomic distance is much less than the resonant wavelength *λ*_*a*_. Since the complete Hilbert space of single excitation involves *N* possible constructions of the bare states $|e{\rangle }_{\mu }|g{\rangle }^{(N-1)}\equiv |{\psi }_{\mu }\rangle$, then the remaining *N* − 1 nonsymmetric states can be either super- or subradiant, depending on the atomic distributions. To systematically study and access those states, we have considered to use a phase-imprinting method^37,38,41^ on a one-dimensional atomic array, which prepares the system into a De Moivre state:

2 $$|{{\rm{\Phi }}}_{m}\rangle =\frac{1}{\sqrt{N}}\,\sum _{\mu =1}^{N}\,{e}^{i{{\bf{k}}}_{L}\cdot {{\bf{r}}}_{\mu }}{e}^{i\frac{2m\pi }{N}(\mu -1)}|{\psi }_{\mu }\rangle ,$$

with *m* ∈ [1, *N*], whose orthogonality is guaranteed by De Moivre’s theorem. This phase imprinting method dynamically controls the linearly increased atomic phases either by a gradient Zeeman field or a gradient Stark field pulse. We note that the form of De Moivre states in equation (2) with **r**_*μ*_ → 0 is first constructed in the degenerate subspace of a small sample^59^.

While this method is simple, practically it, however, demands a large field gradient or long interaction time when the atomic array is short and/or the inter-atomic separation is small. Alternatively, by deforming the atomic array into a ring, this linearly increasing phase can be easily and exactly imprinted by light with a quantized orbital angular momentum (OAM). Thus, we consider a Laguerre-Gaussian (LG) beam in the paraxial approximation^60–62^,

3 $${U}_{p}^{l}(r,\varphi ,z)=\frac{C\hat{\varepsilon }}{w(z)}{(\frac{\sqrt{2}r}{w(z)})}^{|l|}{L}_{p}^{|l|}(\frac{2{r}^{2}}{{w}^{2}(z)}){e}^{-{[r/w(z)]}^{2}-il\varphi }{e}^{-i{k}_{L}z+i\psi (z)-i{k}_{L}{r}^{2}/[2R(z)]},$$

where $\hat{\varepsilon }$ denotes the polarization, $w(z)={w}_{0}\sqrt{1+{(z/{z}_{R})}^{2}}$ is Gaussian beam width with the beam waist *w*_0_, and ${z}_{R}=\pi {w}_{0}^{2}/\lambda$ is Rayleigh range. We denote the normalization constant as *C*, and ${L}_{p}^{|l|}$ is the associated Laguerre polynomials with radial mode numbers *p*. The Guoy phase is $\psi (z)=(2p+|l|+1)\,{\tan }^{-1}(z/{z}_{R})$ and the radius of curvature is *R*(*z*) = *z*[1 + (*z*_*R*_/*z*)^2^]. This paraxial approximation of LG beams is valid when $f=\lambda /(2\pi {w}_{0})\ll 1$, and therefore, the spatially varying Guoy phase and [${e}^{-i{k}_{L}{r}^{2}/[2R(z)]}$] of the wavefront vanish respectively at *z* = 0 and infinite *R*(*z* = 0). Under this circumstance, the HPI states can be prepared with a phase of *e*^*ilϕ*^ along the azimuthal direction. In our preparation scheme of various ring structures, we choose excitation photons with LG modes with *p* = 0 and assume they uniformly excite the atoms on absorption, similar to the timed Dicke state preparation^63^. We denote LG modes with zero radial index (*p* = 0) as

4 $${u}_{p=0}^{l}(r,\varphi )=\frac{C\hat{\varepsilon }}{{w}_{0}}{(\frac{\sqrt{2}r}{{w}_{0}})}^{|l|}{e}^{-{[r/{w}_{0}]}^{2}-il\varphi },$$

where we have replaced ${L}_{p=0}^{|l|}(x)=1$ for arbitrary *x*.

**Figure 1:**
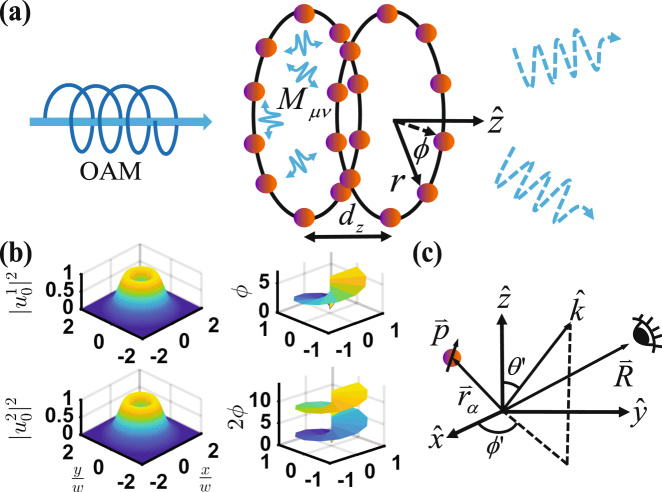
Schematic helical-phase-imprinted state preparation and far-field detection. (**a**) A single-photon source with orbital angular momentum (OAM) is absorbed by the atoms sitting on the stacked rings along $\hat{z}$ (two ring arrays are shown here for illustration). The atomic system is then prepared into one of the super- or subradiant states, depending on OAM of light. In the process of spontaneous decay, the resonant dipole-dipole interaction *M*_*μν*_ couples *μ*^th^ and *ν*^th^ atoms on the stacked rings, and scatters the light collectively depending on the ring geometry of radius *r*, *ϕ*, and inter-ring distance *d*_*z*_. (**b**) Typical light intensity (normalized) with OAM of Laguerre-Gaussian modes ${u}_{0}^{l}(r,\varphi )$ and associated helical phases *e*^*ilϕ*^, for some beam waist radius *w*. (**c**) A far-field observer at $\overrightarrow{R}$ sees the scattered light from a dipole $\overrightarrow{p}$ at ${\overrightarrow{r}}_{\alpha }$ in 4*π* solid angle of mode $\hat{k}$ characterized by *θ*′ and *ϕ*′.

For *N* atoms sitting on a single ring with a constant separation between their nearest neighbors, the light propagating along the axis of the ring imprints the phase of *ϕ* = 2*πl*(*μ* − 1)/*N* on the atoms. A ${u}_{0}^{l}$ photon absorbed by the ring array thus forms exactly the state of |Φ_*m* = *l*_〉 in equation (2). In Fig. 1, we show two stacked rings for an illustration of preparing such helical-phase-imprinted (HPI) states with a ${u}_{0}^{l}$ photon, and the far-field observation of these states. For the multiply-stacked rings along $\hat{z}$, HPI states in general can be expressed as

5 $$|{{\rm{\Phi }}}_{l}{\rangle }_{{\rm{HPI}}}=\frac{1}{\sqrt{N}}\,\sum _{{\mu }_{z}=1}^{{N}_{z}}\,\sum _{{\mu }_{\varphi }=1}^{{N}_{\varphi }}\,{e}^{i{{\bf{k}}}_{L}\cdot {{\bf{r}}}_{\mu }}{e}^{i\frac{2l\pi }{{N}_{\varphi }}({\mu }_{\varphi }-1)}|{\psi }_{\mu }\rangle ,$$

where *N* = *N*_*z*_*N*_*ϕ*_ for a number of *N*_*z*_ stacked rings, with *N*_*ϕ*_ atoms in each ring. The atomic position index *μ* is implicitly (*μ*_*z*_ − 1)*N*_*ϕ*_ + *μ*_*ϕ*_, which labels the traveling phase by the excitation field on |*ψ*_*μ*_〉. The multiply-stacked rings allow a larger optical depth and stronger light-matter interactions, and thus increase the absorption efficiency. For the stacked rings along $\hat{r}$, forming a concentric structure in a two-dimensional plane, we can substitute *N*_*z*_ and *μ*_*z*_ with *N*_*r*_ and *μ*_*r*_ respectively in equation (5).

Below we investigate the emission patterns of HPI subradiant states, assuming they are excited and prepared genuinely. The efficiency, on the other hand, can be enhanced by using heralded single photon source or post selections. We note that non-perfect technical issues, such as nonparaxial conditions and the nonuniform spatial profile of an LG beam for multiply-stacked rings, compromise either the fidelity or efficiency of HPI state preparation, but can still be optimized by, for instance, using beams with a longer Rayleigh range or equivalently larger beam waist and by optimizing the alignment^64^.

## Light Scattering from HPI Subradiant States

In this section, we consider the resonant dipole-dipole interaction present in the dissipation of HPI states. This long-range interaction^23,24^ arises from rescattering photons between atoms in the spontaneous emissions, and is the mechanism for superradiance or subradiance when atoms are close to each other. The explicit forms of the interaction and the far-field emission patterns of HPI states are derived in Methods respectively. Below we investigate the emission patterns for various atomic ring structures. We note that the angular distributions of the superradiant states have been recently investigated in linear chains^65–68^.

### Two-atom case

**Figure 2:**
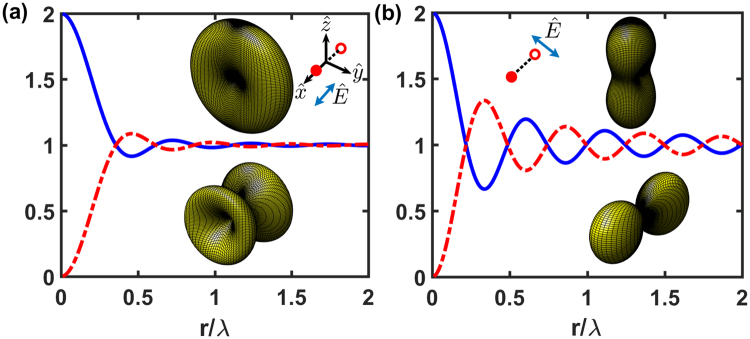
Eigenvalues and far-field property Ω_*l*_(*θ*, *ϕ*) for two atoms sitting on the $\hat{x}$ axis. We show the real part of the rescaled eigenvalues, −*λ*_1,2_/(Γ/2), in the case of two atoms separating by 2*r*, for the eigenstates of *l* = 1 (red dashed line, subradiant) and 2 (blue solid line, superradiant) with (**a**) $\hat{x}$ and (**b**) $\hat{y}$ polarized light excitations $\hat{E}$. Specific three-dimensional plots of Ω_*l*_(*θ*, *ϕ*) at *r*/*λ* = 0.15 for the super- and subradiant states are illustrated respectively in the upper and lower parts of the graphs. Empty and filled circles indicate the ground and excited atoms, one of the bare states |*ψ*_*μ*_〉. The fitted exponential decay rates (in units of Γ/2) for super- and subradiant states are Γ_*f*_ = (a) 1.5 and 0.51, (b) 1.1 and 0.9, respectively.

We first analyze the case of two atoms sitting on a ring with a radius *r* and excited by single photon carrying an OAM, *lħ*. Define the far-field scattering intensity ${{\rm{\Omega }}}_{l}(\theta ,\varphi )\equiv {\langle {\overrightarrow{E}}^{\ast }({\bf{R}},t)\overrightarrow{E}({\bf{R}},t)\rangle }_{l}/{I}_{0}(t)$, we use equation (17) in Methods for two atoms on the *x*-axis with an $\hat{x}$ polarized excitation, and we obtain

6 $${{\rm{\Omega }}}_{l}(\theta ,\varphi )=(1-{\sin }^{2}\,\theta \,{\cos }^{2}\varphi )[2+2\,\cos \,(\mathrm{2|}{{\bf{k}}}_{L}|r\,\sin \,\theta \,\cos \,\varphi +l\pi )],$$

which corresponds to the case shown in Fig. 2(a), where two atoms are aligned parallel to the polarization of the excitation field. For the case where two atoms are aligned perpendicular to the polarization which is shown in Fig. 2(b), the factor in equation (6), $1-{\sin }^{2}\,\theta \,{\cos }^{2}\,\varphi$, is replaced by ${\sin }^{2}\,\theta \,{\sin }^{2}\,\varphi$. Note that |**k**_*R*_| = |**k**_*L*_|, and different light polarizations result in different coupling strengths in equations (11) and (12) of Methods, and thus *I*_0_(*t*), due to different eigenvalues *λ*_*m*_ (see Methods). When *r* → 0, Ω_*l*_(*θ*, *ϕ*) $\propto$ [1 + (−1)^*l*^], which indicates that the excitation beam with odd OAM is not scattered at all in this extreme limit. According to equation (5), this specific HPI state is given as

7 $$|{{\rm{\Phi }}}_{l}{\rangle }_{{\rm{HPI}}}=\frac{1}{\sqrt{2}}({e}^{i{{\bf{k}}}_{L}\cdot {{\bf{r}}}_{1}}|{\psi }_{1}\rangle +{e}^{i{{\bf{k}}}_{L}\cdot {{\bf{r}}}_{2}}{e}^{il\pi }|{\psi }_{2}\rangle ),$$

which is a superradiant (subradiant) state for even (odd) *l*. The superradiant intensity for single photon scattering in the forward direction has a maximal Ω_*l*_(*θ* = 0) = 2 which is proportional to *N*^2^/2, as that in the half-excited spin models^21^. For single spin excitation in the *N* spin-1/2 model, the photon emission intensity is proportional to (*l*_*m*_ + *m*)(*l*_*m*_ − *m* + 1) = *N* in the Dicke’s eigenstates with a total quantized angular momentum *l*_*m*_*ħ* = *Nħ*/2 and magnetization *m* ≡ (*N*_↑_ − *N*_↓_)/2 = 1 − *N*/2^21^.

In Fig. 2, we show the eigenvalues and far-field property Ω_*l*_(*θ*, *ϕ*) for two atoms separating by 2*r*. The eigenvalues can be solved analytically from the coupling matrix $\hat{M}$ introduced in Methods, which are

8 $${\lambda }_{\mathrm{1,2}}=-\,\frac{{\rm{\Gamma }}}{2}\pm [\frac{{F}_{12}(\xi )-i2{G}_{12}(\xi )}{2}],$$

where Γ is the natural decay rate (linewidth) of the excited-ground transition, and *ξ* = 2|**k**_*L*_|*r*. The rescaled real part of the eigenvalues in Fig. 2, which are decay constants, approach 2 and 0 as *r* → 0, representing the super- and subradiant modes of the radiation. For larger *r*, the eigenvalues asymptotically converge to 1, corresponding to the regime of non-interacting (independent) emitters. Specific far-field property is chosen at *ξ* = 0.3*π*, which shows a forward-backward scattering along the excitation propagation direction, $\hat{z}$, and a transverse scattering for the super- (*l* = 2) and subradiant (*l* = 1) modes, respectively. Note that the $\hat{x}$ and $\hat{y}$ polarized excitations correspond to the head-to-tail and parallel polarization configurations, respectively, and the former (latter) suppresses light scattering in the $\hat{x}$ ($\hat{y}$) direction, which can be seen from equation (6) that the scattered light vanishes at *θ* = *π*/2 and *ϕ* = 0 (*π*/2). The head-to-tail polarizations also allow strong scattering in the superradiant mode in the direction transverse to the polarization orientation. On the contrary, with the parallel polarizations, the collective scattering rate along the $\hat{x}$ axis is suppressed to ~0.35 times the maximum scattering rate. This reflects the destructive interference of parallel polarized (virtual) photons from two atoms, which diminishes as *r* → 0, where Ω_*l*_(*θ*, *ϕ*) of these two polarization configurations restores the rotational symmetry of the superradiance case in Fig. 2(a), by taking *ϕ* → *ϕ* + *π*/2.

The subradiant modes, on the contrary, preserve the scattering patterns in Fig. 2 at *r*/*λ* $\lesssim$ 0.35 and 0.25 for head-to-tail and parallel polarizations respectively. This range of *r* can be estimated by $\mathrm{2|}{{\bf{k}}}_{L}|r\,\sin \,\theta \,\cos \,\varphi \approx \pi \mathrm{/2}$ in equation (6), which indicates the phase slip of half of *lπ* (*l* = 1 for the subradiant mode). The angles can be chosen as *θ* = *π*/4 (*π*/2) and *ϕ* = 0 at the maximal scattering of the head-to-tail (parallel) polarizations in the small *r* limit. This estimation also reflects on the qualitative changes of the eigenvalues in Fig. 2, which start to oscillate around *λ*_1,2_ = −Γ/2. As *r* increases and passes the estimated range of 0.35*λ* or 0.25*λ*, a scattering transverse to forward/backward direction also appears in the superradiant modes, and the directionality of them disappears. In the range of $2r\gtrsim \lambda$, the clear phenomena of super- and subradiance disappear.

### Single ring

For the geometry of *N* atoms sitting on a single ring with equal arc lengths, it is equivalent to an *N*-sided regular polygon. When $N\gg 1$, the far-field scattering pattern from atoms forming a regular polygon approaches that of a ring. Before we investigate the scattering of the many-body subradiant states in a single ring with a large *N*, we first study the case of three and four atoms, which form regular triangle and square respectively.

**Figure 3:**
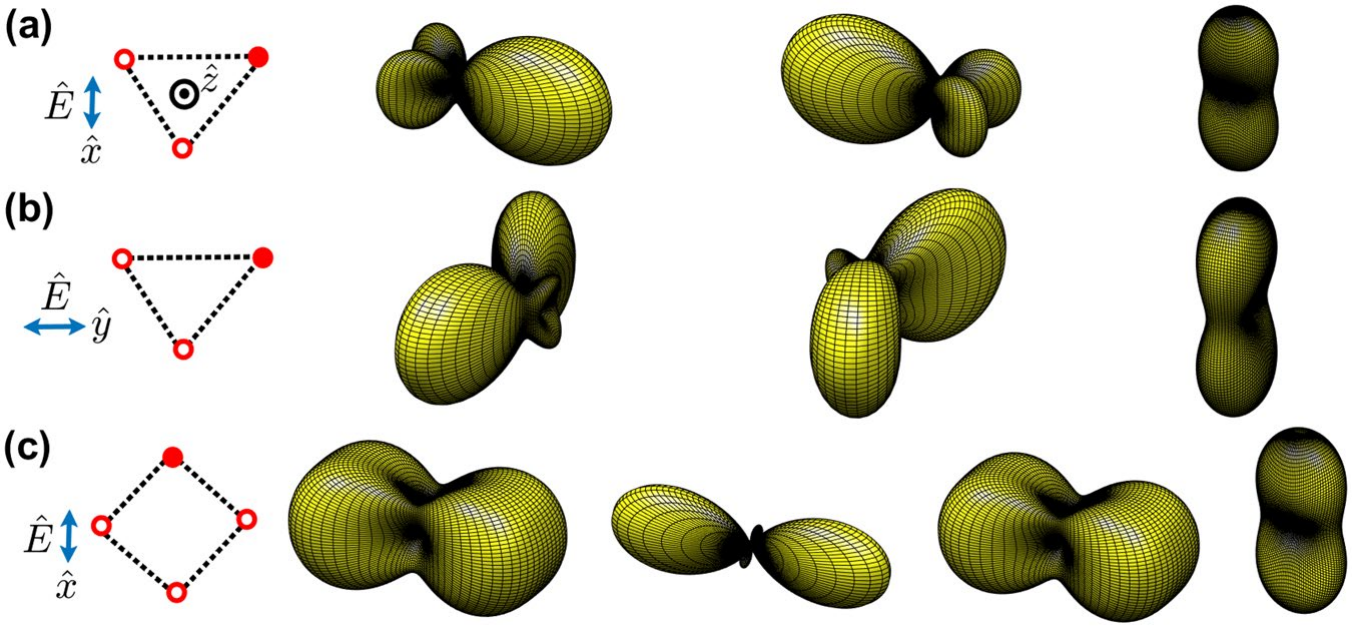
The far-field Ω_*l*_(*θ*, *ϕ*) for three and four atoms sitting on a single ring. The atoms in top view form an (**a**) $\hat{x}$ and (**b**) $\hat{y}$ polarized triangular, and (**c**) $\hat{x}$ polarized square structures with the modes of *l* = 1 − *N* respectively, where we choose *r*/*λ* = 0.2. Subradiant modes (*l* = 1 to *N* − 1) and superradiant modes (*l* = *N*) show directional transversal and forward-backward scatterings respectively. Note that Ω_*l*_(*θ*, *ϕ*) of $\hat{y}$ polarized square preserves the *C*_4_ rotational symmetry to the case of (**c**), and the viewing angles are the same as Fig. 2. Again empty and filled circles represent the ground and excited atoms, which displays one of the bare states |*ψ*_*μ*_〉. The fitted exponential decay rates for three- and four-atom rings (in units of Γ/2) are, Γ_*f*_ = (**a**) 0.72, 0.72, 1.9, (**b**) 0.7, 0.7, 1.9, (**c**) 0.75, 0.13, 0.75, and 2.51 respectively.

In Fig. 3(a,b), we show the far-field scattering of the HPI states, Ω_*l*_(*θ*, *ϕ*)’s with *l* = 1–3, for three atoms which are in a regular-triangle configuration and are excited by $\hat{x}$ and $\hat{y}$ polarized light, respectively. As expected, the superradiant modes for both polarizations, corresponding to *l* = 3, show directional forward-backward scatterings along $\hat{z}$. In contrast, for the subradiant modes with *l* = 1 and 2, the forward-backward scatterings are suppressed, and light is preferentially emitted into directions perpendicular to both *k* vector and the polarization of the incoming photon, which makes the $\hat{x}$ − $\hat{z}$ and $\hat{y}$ − $\hat{z}$ planes the nodal planes of Ω_*l*_’s for $\hat{x}$ and $\hat{y}$ excitations, respectively.

In Fig. 3(c), we study the case with four atoms forming a square. The *l* = 2 subradiant mode, possessing the lowest decay rate, is more directional than the modes with *l* = 1 and 3 which have the same Ω_*l*_(*θ*, *ϕ*). Furthermore, in this specific structure, two perpendicularly excited polarizations result in the same pattern of the far-field scattering which preserves the *C*_4_ rotational symmetry in *ϕ*. This *C*_4_ symmetry also applies to all the number of atoms *N* = 4*n* with integers *n*. We note that the far-field scatterings of the *l* = 1 and 3 modes are the same due to the symmetry of *l* → −*l*, which will be further explained in Methods.

**Figure 4:**
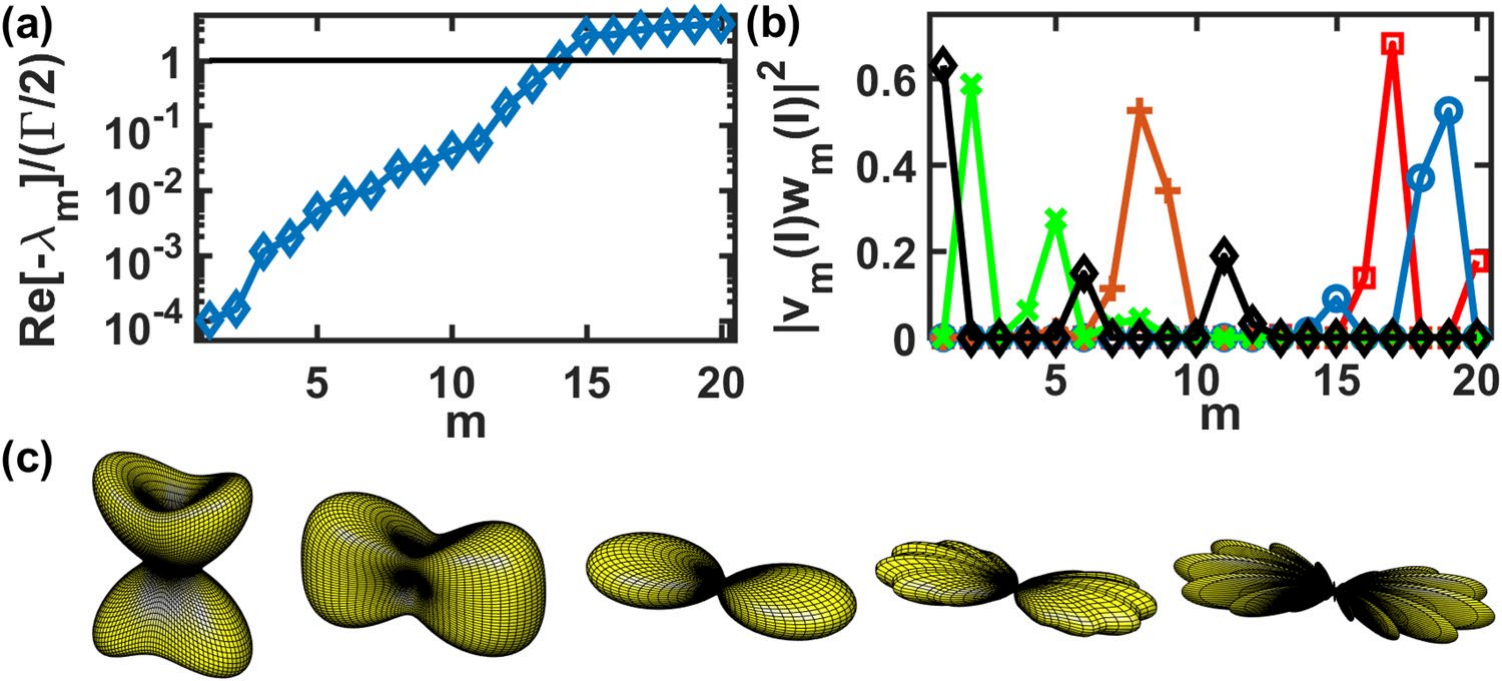
The decay rates, normalized weightings, and Ω_*l*_(*θ*, *ϕ*) of a single ring with *N* = 20 and *r*/*λ* = 0.5. (**a**) The spontaneous decay rates shown in ascending order and logarithmic scale, which are derived from the real part of the eigenvalues *λ*_*m*_. (**b**) The normalized weightings on the eigenmodes for *l* = 1, 2, 5, 9, and 10 (○, □, +, ×, and ◊ respectively), and (**c**) the corresponding Ω_*l*_(*θ*, *ϕ*) (left to right), in the same viewing angle of Fig. 2. The fitted exponential decay rates for *l* = 1, 2, 5, 9, and 10 (in units of Γ/2), Γ_*f*_ = 5.43, 4.77, 0.024, 1.3 × 10^−3^, and 5 × 10^−4^, respectively.

For many atoms on a single ring, we use *N* = 20 as an example and show the calculation results in Fig. 4. In this configuration, *C*_4_ rotational symmetry sustains, and therefore, Ω_*l*_(*θ*, *ϕ*) are the same for two linear polarizations. Figure 4(a) shows the calculated eigenmodes, with 14 subradiant and 6 superradiant modes, as the 14th mode is just below the line of *λ*_*m*_ = −Γ/2. The lowest subradiant eigenmode is 10^−4^ times the natural decay rate for *r*/*λ* = 0.5, which can be further reduced as *r* decreases. We selectively plot several representative HPI states in Fig. 4(b), in which each only occupies a few eigenmodes with significant weightings. It is seen that HPI states with *l* = 1 and 2 are superradiant, while that with *l* = 5, 9, and 10 are subradiant. In Fig. 4(c), the Ω_*l*_(*θ*, *ϕ*) is plotted accordingly for the selected modes in 4(b). Except for the symmetric superradiant state of *l* = *N*, which has a clear forward-backward scattering similar to the previous cases of few atoms, the other superradiant modes, e.g., with *l* = 1 and 2, also show an oblique and transverse scatterings respectively. As *l* increases toward the most subradiant modes (*l* = 10), a clear side scattering at the right angles emerges, and this most subradiant HPI state further breaks Ω_*l*_(*θ*, *ϕ*) into 10 concentric and flat lobes, each with a good directionality.

We note that Ω_*l*_(*θ*, *ϕ*) = Ω_−*l*_(*θ*, *ϕ*) = Ω_*N*−*l*_(*θ*, *ϕ*) for even number of the atoms, which is also true for odd number of atoms at *r* = 0. For small *r*, Ω_*l*_(*θ*, *ϕ*) of can be approximately reduced to the sum of imprinted helical phases ${e}^{i2l\pi ({\beta }_{\varphi }-{\alpha }_{\varphi })/{N}_{\varphi }}$. This gives in general a sum of cosine functions without the detail spatial phases from the atomic distributions of **r**_*αβ*_; therefore, Ω_*l*_(*θ*, *ϕ*) is the same for *l*th and (*N* − *l*)th HPI states. For a finite *r*, only even number of the atoms *N* sustains the symmetry of *l* → −*l* in Ω_*l*_(*θ*, *ϕ*). This can be seen from the pairwise and spatial phase contributions of **k**_*R*_ · **r**_*αβ*_, and we explain in more details in Methods.

### Stacked rings

**Figure 5:**
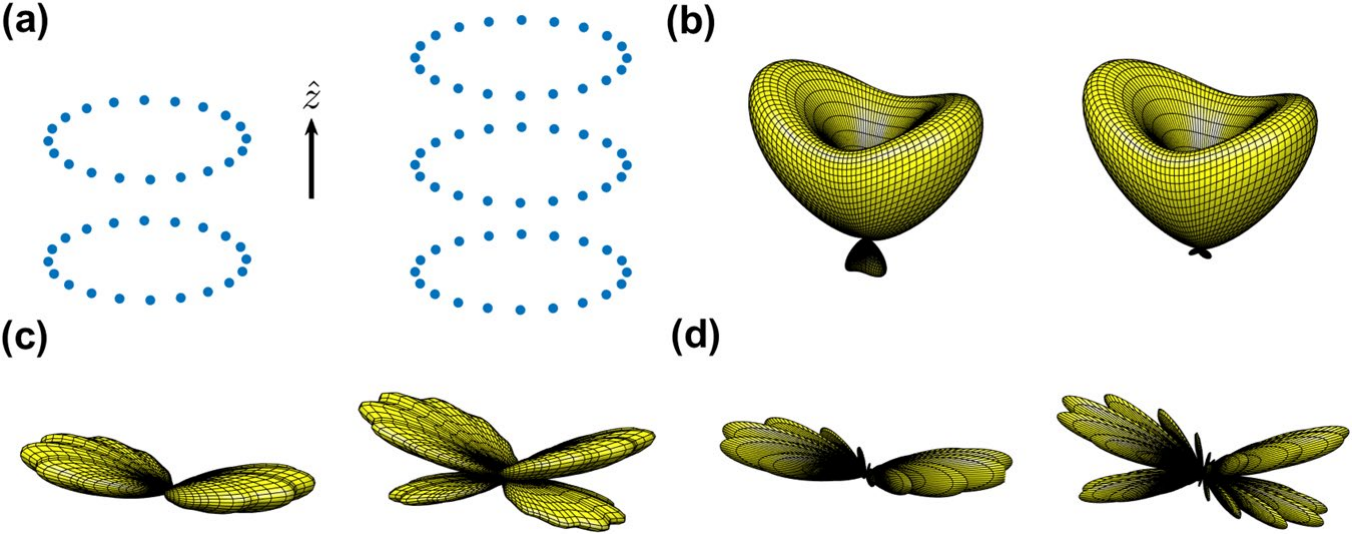
Schematics of $\hat{z}$-stacked ring arrays and their far-field properties Ω_*l*_(*θ*, *ϕ*), with *r*/*λ* = 0.5. For the stacked ring arrays with *N*_*ϕ*_ = 20 in (**a**), Ω_*l*_(*θ*, *ϕ*)’s of the HPI states of two and three $\hat{z}$-stacked rings with *d*_*z*_/*λ* = 0.35 are illustrated for (**b**) *l* = 1 (superradiant), (**c**) *l* = 9 (subradiant), (**d**) *l* = 10 (subradiant), which exhibit enhancement on the forward scattering as the number of rings increases. The viewing angles are the same as Fig. 2.

Atomic rings can be put into stacked configurations to further enhance the cooperative effects. In the following, we investigate $\hat{z}$- and planer concentric-stacking (referred as $\hat{r}$-stacking here), in which the assemblies are integrated along either the axial or the radial directions, respectively. Figure 5(a) shows schematically two and three $\hat{z}$-stacking rings, in which the inter-ring distance is characterized by the parameter *d*_*z*_. For the HPI superradiant mode with *l* = 1, as in Fig. 5(b), the forward scattering is enhanced as number of rings increases, which breaks the symmetry of forward-backward scattering in Fig. 4(c). For the subradiant mode in Fig. 5(c,d), an oblique scattering toward the forward direction is enhanced as more atoms are integrated along the $\hat{z}$ direction. As we stack up more rings, the transverse scattering at *θ* = *π*/2 in Fig. 4(c) can reappear as well. Therefore, the far-field scattering pattern can be greatly tuned by varying *r*, *d*_*z*_ ($\lesssim$*λ*), and the number of the stacked rings, which controls the intra- and inter-ring phase interferences.

**Figure 6:**
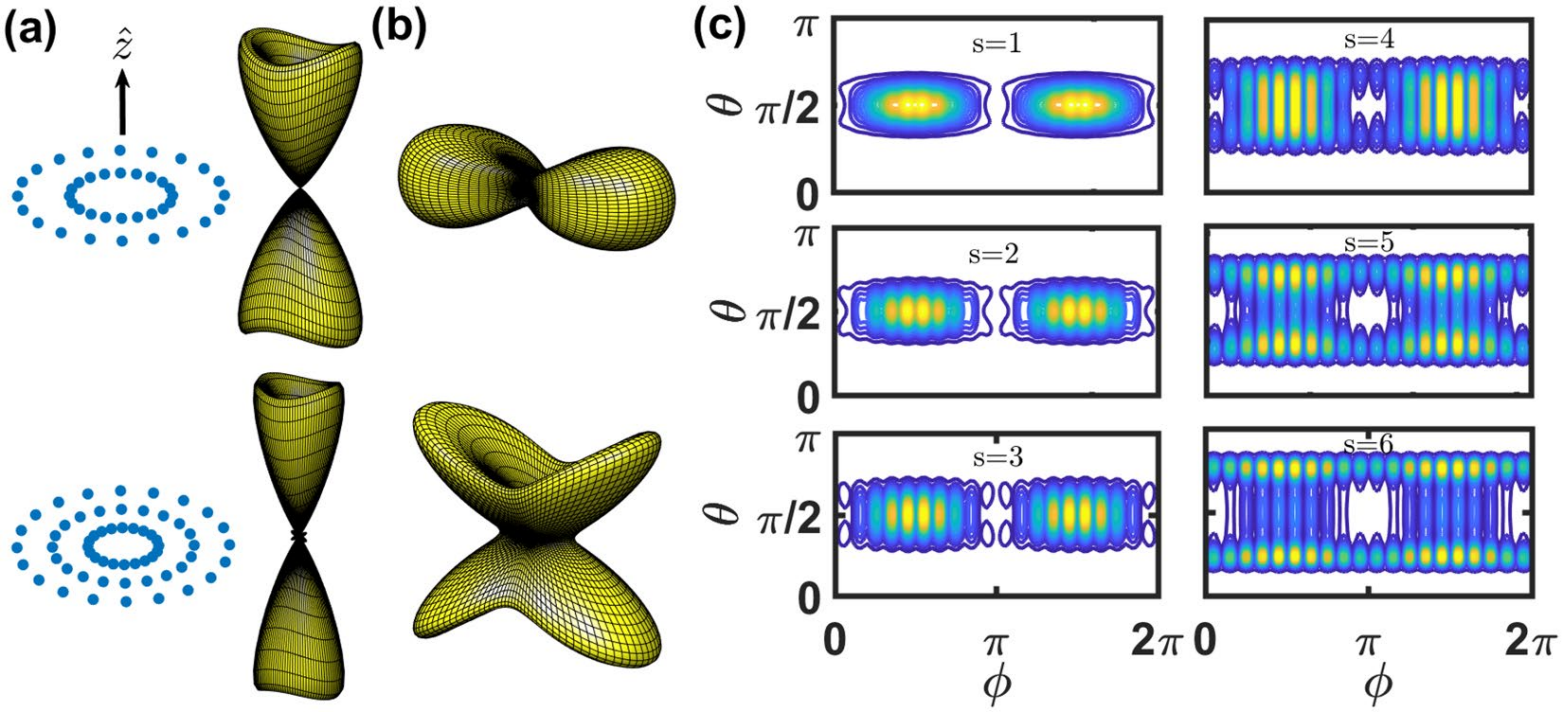
Schematics and the far-field scattering Ω_*l*_(*θ*, *ϕ*) for *s* $\hat{r}$-stacked rings. (**a**) Schematic of $\hat{r}$-stacked rings with the Same *N*_*ϕ*_ as that in Fig. 5 in each ring, with equal inter-ring distance *d*_*r*_. The Ω_*l*_(*θ*, *ϕ*)’s of the HPI states of two (upper) and three (lower) $\hat{r}$-stacked rings are illustrated for (**a**) *l* = 1 (superradiant), (**b**) *l* = 5 (sub- and superradiant for two and three rings respectively), and cross-sectional plots for (**c**) *l* = 9 (subradiant) excitations. The scattering intensity shows narrowing (localization) in polar angle *θ* for *l* = 1 modes while in both *θ* and *ϕ* for modes with *l* = 9 as number of rings increases. The splitting and further narrowing in *θ* for the states with *l* = 9, as seen in (**c**) for *s* from 1 to 6, indicating a crossover from the sub- to the superradiant modes. This crossover also appears in (**b**). The fitted exponential decay rates for *s* = 1–6 (in units of Γ/2), Γ_*f*_ = 1.3 × 10^−3^, 7.7 × 10^−3^, 0.019, 1.4, 1.35, and 1.36, respectively.

In Fig. 6, we study the HPI states of *s* $\hat{r}$-stacking rings with *l* = 1 (superradiant), *l* = 5 (sub- and superradiant for *s* < 3), and *l* = 9 (subradiant for *s* < 3; sub- and superradiant for 4 < *s* < 6). In this configuration, the inter-ring distance is characterized by *d*_*r*_. Compared to the super- and subradiant modes in a single ring shown in Fig. 4(c), we find narrowing effects on Ω_*l*_(*θ*, *ϕ*) for both *θ* and *ϕ* directions. For the *l* = 1 mode shown in Fig. 6(b), as the number of rings *s* is increased, far-field radiation is elongated toward forward and backward directions with narrower distributions in *θ* for each lobe. For *l* = 5 mode which is shown in Fig. 6(b), with the core-ring radius *r*_*c*_ = 0.5*λ*, inter-ring distance *d*_*r*_ = *r*_*c*_, and the number of rings *s* = 3, Ω_*l*_(*θ*, *ϕ*) splits into two lobes, bending toward forward and backward directions, similar to that in Fig. 6(a). This drastic change of scattering directions also accompanies with a crossover from sub- to superradiance. For the subradiant modes with *l* = 9 in Fig. 6(c), we also see localization of scattering in *ϕ* direction as *s* increases to 3, with the number of narrow lobes equal to *N*_*ϕ*_ = 20. For *s* = 4 in Fig. 6(c), we see a noticeable broadening in *θ* direction, which again exhibits a crossover from the sub- to the superradiance. The mode at *s* = 6 becomes strong superradiant, but in contrast to the case of *l* = *N*_*ϕ*_ (equivalent to *l* = 0) which exhibits simply forward and backward scattering along $\hat{z}$, it further breaks the continuous symmetry in the *ϕ* direction and lead to 20 narrow superradiant lobes. Similar behaviors are seen in superradiant modes with *l* = 8. We note that the equivalence of maximum number of narrowed superradiant lobes and the number of atoms on each ring for a large *l* ≤ *N*_*ϕ*_/2 (*l* = 8, 9, 10) is also a signature of the HPI states of $\hat{r}$-stacking rings. In contrast, for the $\hat{z}$-stacking rings, this feature only appears in the case with *l* = *N*_*ϕ*_/2, as shown in Figs 4(c) and 5(c).

### Circular polarization

**Figure 7:**
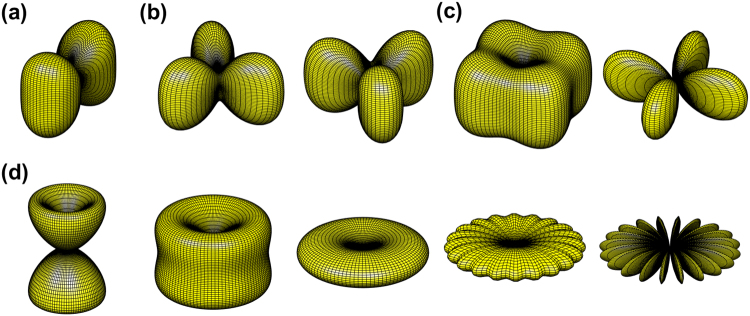
Far-field scattering from circularly polarized excitations. The Ω_*l*_’s of the HPI subradiant states are shown for (**a**) two-atom (*l* = 1), (**b**) three-atom (*l* = 1 and 2), and (**c**) four-atom cases (*l* = 1 and 2), at *r*/*λ* = 0.2. (**d**) The far-field scatterings of a single ring (same *N* and *r* for Fig. 4) with a circularly polarized excitation with *l* = 1, 2, 5, 9, and 10 are illustrated horizontally. The fitted exponential decay rates (in units of Γ/2), Γ_*f*_ = (**a**) 0.7, (**b**) 0.57 for both modes, (**c**) 0.7, 0.11, (**d**) 4, 3.3, 0.018, 7 × 10^−8^, and 3.5 × 10^−9^, respectively.

For the HPI states excited with circular polarization, [$(\hat{x}\pm i\hat{y})/\sqrt{2}$], we expect more symmetric scattering patterns than with linear polarizations, and this is indeed the case as seen in Fig. 7. In this figure, we display the Ω_*l*_’s of the HPI subradiant states with *l* = 1 and 2 for few-atom cases, and that for many atoms with parameters adopted in a single ring structure in Fig. 4. For any uniform polarizations in general, ${(\hat{{\bf{R}}}\cdot \hat{{\bf{p}}})}^{2}$ in equation (17) can be replaced by $|\hat{{\bf{R}}}\cdot \hat{{\bf{p}}}{|}^{2}$, and as such, the handedness of the circular polarization does not matter to Ω_*l*_(*θ*, *ϕ*). For the two-atom case, the scattering property can be derived by substituting the prefactor of equation (6), ${\sin }^{2}\,\theta \,{\cos }^{2}\,\varphi$, by ${\sin }^{2}\,\theta \mathrm{/2}$. Similar to the case with linear polarizations in Fig. 2, scattering is suppressed on the $\hat{y}$ − $\hat{z}$ plane but maximized on the $\hat{x}$ − $\hat{z}$ plane, which can be identified in Fig. 7(a). This is exactly the sum of the far-field scattering of HPI state for two linear-polarizations with *l* =  = 1 in Fig. 2. For three and four atoms, Ω_*l*_(*θ*, *ϕ*) of the subradiant modes peak at discrete azimuthal angles *ϕ* = *ϕ*_*s*_ + 2*π*/*N*, preserving the *C*_*N*_ rotational symmetry for *ϕ*, where *ϕ*_*s*_ is the offset of the angle, depending on which subradiant mode we consider in Fig. 7(b,c).

For a single ring with *N* = 20 atoms, the HPI states go from super- to sub-radiant modes as *l* is increased, in which scattering in the forward/backward directions diminishes while the scattering in the transverse direction grows. For subradiant modes with *l* = 9 and 10, Ω_*l*_(*θ*, *ϕ*) breaks into 20 radiation lobes with narrow distribution in *ϕ*. In contrast to the similar effect seen in Figs 4, 5 and 6, circular polarization gives higher azimuthal rotational symmetry. This rotational *C*_*N*_ symmetry also applies to the cases of $\hat{z}$- and $\hat{r}$-stacking rings in Fig. 5. In the $\hat{r}$-stacking configurations, narrowing effects in *θ* and *ϕ* also appear, similar to that in Fig. 6(c).

We note that in general, elliptically polarized light represents all possible uniform polarizations of field excitations, and therefore, linear and circular polarizations are the special cases of elliptical ones. In terms of a normalized Jones vector, an elliptical polarization can be expressed as ($\cos \,{\theta }_{a}\hat{x}+\,\sin \,{\theta }_{a}{e}^{i{\theta }_{e}}\hat{y}$) where *θ*_*a*_ and *θ*_*e*_ are two angles to characterize the strengths and phases of the polarizations in $\hat{x}$ − $\hat{y}$ plane. The vector of elliptical polarization traverses a rotated ellipse with an orientation angle *α* from the $\hat{x}$-axis determined by tan *α* = tan *θ*_*a*_ cos *θ*_*e*_. Therefore, (*θ*_*e*_ = 0) and (*θ*_*e*_ = ±*π*/2, *θ*_*a*_ = *π*/4) correspond to linear and circular polarizations respectively. For other angles, elliptical polarizations should generate the emission patterns with unequal superpositions of $\hat{x}$- and $\hat{y}$-polarized cases. Here and in the previous section, we show the results of circular and linear polarizations only, which should be sufficient and representative.

### Radial and azimuthal polarizations

Finally we study the scattering properties from the radially and azimuthally polarized excitations^64^, which allows us to further manipulate the local orientations of the atomic dipoles. The resonant dipole-dipole interactions in equations (11) and (12) can be straightforwardly generalized by replacing the term [1 − ${(\hat{{\bf{p}}}\cdot {\hat{r}}_{\mu \nu })}^{2}$] with [$({\hat{{\bf{p}}}}_{\beta }^{\ast }\cdot {\hat{{\bf{p}}}}_{\alpha })$ − $({\hat{{\bf{p}}}}_{\beta }^{\ast }\cdot {\hat{r}}_{\alpha \beta })$ $({\hat{{\bf{p}}}}_{\alpha }\cdot {\hat{r}}_{\alpha \beta })$] and correspondingly the term [1 − $\mathrm{3(}\hat{{\bf{p}}}\cdot {\hat{r}}_{\mu \nu }{)}^{2}$] with [$({\hat{{\bf{p}}}}_{\beta }^{\ast }\cdot {\hat{{\bf{p}}}}_{\alpha })$ − $\mathrm{3(}{\hat{{\bf{p}}}}_{\beta }^{\ast }\cdot {\hat{r}}_{\alpha \beta })$ $({\hat{{\bf{p}}}}_{\alpha }\cdot {\hat{r}}_{\alpha \beta })$]. Similarly, the far-field Ω_*l*_ of equation (17) can also be generalized by replacing [1 − ${(\hat{{\bf{R}}}\cdot \hat{{\bf{p}}})}^{2}$] with [$({\hat{{\bf{p}}}}_{\beta }^{\ast }\cdot {\hat{{\bf{p}}}}_{\alpha })$ − $({\hat{{\bf{p}}}}_{\beta }^{\ast }\cdot \hat{{\bf{R}}})$ $({\hat{{\bf{p}}}}_{\alpha }\cdot \hat{{\bf{R}}})$] and summing over indices *α* and *β*.

**Figure 8:**
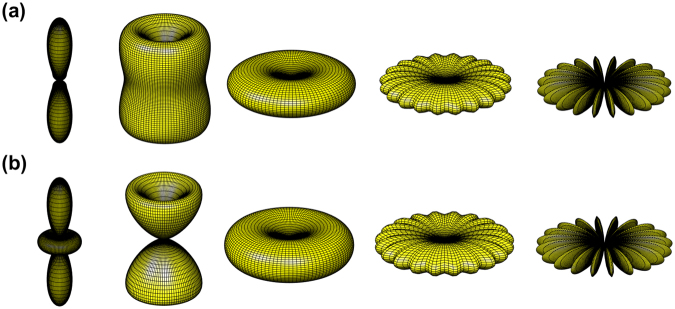
Far-field scattering from radially and azimuthally polarized excitations. The Ω_*l*_’s of the HPI super- and subradiant states are plotted for a single ring with the same *N* and *r* of Fig. 4, which are excited by (**a**) radial and (**b**) azimuthal polarizations. The modes of *l* = 1, 2, 5, 9, and 10 are illustrated from left to right. The corresponding fitted decay rates for these modes (in units of Γ/2), Γ_*f*_ = (**a**) 2.3, 3.5, 0.098, 1.2 × 10^−6^, 7.4 × 10^−8^, (**b**) 3.9, 1.6, 0.074, 1.1 × 10^−6^, 6.9 × 10^−8^, respectively.

The polarizations for the radially and azimuthally excited atoms can be denoted as ${\hat{{\bf{p}}}}_{\alpha }$ = ${\hat{e}}_{r}$ and ${\hat{e}}_{\varphi }$ which are [$\cos \,\varphi (\alpha )\hat{x}$ + $\sin \,\varphi (\alpha )\hat{y}$] and [−$\sin \,\varphi (\alpha )\hat{x}$ + $\cos \,\varphi (\alpha )\hat{y}$] in Cartesian coordinates, respectively. The polarization rotates with an angle *ϕ*(*α*) = 2*πα*_*ϕ*_/*N*_*ϕ*_ which will imprint on the atoms in addition to the OAM phase. Unlike the circular polarizations which have definite spin angular momentum (±1*ħ*) in a photon, the radial and azimuthal polarizations can only relate to the circular ones by ${\hat{e}}_{r}\pm i{\hat{e}}_{\varphi }$ = $(\hat{x}\pm i\hat{y}){e}^{\mp i\varphi }$. This indicates that an equal superposition with ±*π*/2 phase shift between the radial and azimuthal polarizations of light is the same as the left (right)-handed circular polarization with one quanta shift ($\mp \,1\hslash$) of OAM. In Fig. 8, we demonstrate the far-field scattering patterns from radially and azimuthally polarized excitations. Both *l* = 1 HPI states exhibit the superradiant (*l* = *N*_*ϕ*_ or equivalently *l* = 0) scattering patterns with a signature of strong scattering into the forward and backward directions. As *l* increases, we find that the transverse far-field scattering patterns are enhanced while forward/backward directions are suppressed, similar to Fig. 7(d). When *l* ≈ *N*_*ϕ*_/2, the corresponding HPI state is the most subradiant state, and Ω_*l*_ splits into *N*_*ϕ*_ thin slab-like radiation lobes. In this case, we find that all of the patterns from circularly, radially, or azimuthally polarized excitations approach to each other. This is due to both the symmetry of *l* → −*l* in Ω_*l*_(*θ*, *ϕ*) and the discrete rotational symmetry *C*_*N*_ are satisfied. Similarly, the stacked rings along $\hat{z}$ or $\hat{r}$ respectively enhances the forward scattering and the narrowing effect as in Fig. 5 with again an additional *C*_*N*_ symmetry.

## Discussion and Conclusion

In this paper, the requirement for preparing a photon carrying an OAM is relatively easy to fulfill, and a recent experiment showed that an OAM beam with as high as 10,000*ħ* can be generated^69^, thus suggests that our scheme is pragmatic with a scalability up to thousands of atoms. The requirement of reaching strong coupling, *viz*. the inter-atomic separation $d\lesssim 0.5\lambda$, is more stringent but can also be reached. For real atoms, it requires the atomic density comparable to that of an atomic Bose-Einstein condensate (BEC). As such, one can achieve by loading a BEC into a ring trap with a relatively tight radial confinement. On the other hand, it may be even more practical and promising to construct a strong-interacting ring with artificial atoms in solids^70,71^, given the high controllability of the atomic positions nowadays. It is of the reach of current technology to fabricate and construct a ring with *μ*m-sized superconducting devices driven by a microwave field of wavelength *λ* > 500 *μ*m or diamond color centers doped with 40-nm precision and driven by a near-infrared light at *λ* = 750 nm^50^. Both systems allow strong resonant dipole-dipole interactions as *r*/*λ*~0.05. For thermal atomic system^28,72,73^, we can add position fluctuations in our theory to simulate the effect of thermal fluctuations. We expect that the scattering patterns will be smoothed out and the fluctuation will suppress the lifetime^43^ of the HPI subradiant states.

The scattered patterns Ω_*l*_(*θ*, *ϕ*)’s not only provide useful information for light collections but offers far-field scattering fingerprints that can be traced back to the atomic spatial distributions, as well as the polarizations, thus offers one to study light-matter interactions in greater details. For practical applications in quantum memory, as the far-field radiation patterns of the super- and sub-radiant HPI states are very different, one can efficiently collect the transversally scattered light from the subradiant states using a parabolic mirror with the forward and backward scattering blocked. While we have discussed stacking rings along the $\hat{z}$ and $\hat{r}$ directions in this study, there are many other interesting geometries which can be studied, such as atoms arranged in a cylindrical shell with a chirality or even in a torus-like shape, making our scheme a versatile platform for engineering the properties of the HPI many-body states and their emission patterns for quantum optical applications.

Finally, storing and manipulating quantum information using a light with a large OAM in principle allows massive entanglement, which is potentially useful in implementing high-dimensional quantum gates for quantum computation^74^, as well as handling hyper-entangled photons which simultaneously entangle OAM and such as the polarization (spin) degrees of freedom, for higher information capacity^75^.

## Methods

### Lindblad form of dissipation with resonant dipole-dipole interactions

The theoretical analysis for the fluorescence and light scattering is based on the Lindblad forms of the spontaneous emissions. The general spontaneous emission process involves the long-range dipole-dipole interaction^23,24^. This pairwise interaction originates from the rescattering events in the common quantized light field. For an arbitrary quantum operator $\hat{Q}$, the Heisenberg equation in a Lindblad form gives

9 $$\frac{d\hat{Q}}{dt}=-\,i\sum _{\mu \ne \nu }^{N}\sum _{\nu =1}^{N}\,{G}_{\mu \nu }[\hat{Q},{\hat{\sigma }}_{\mu }^{+}{\hat{\sigma }}_{\nu }^{-}]+{{\mathscr{S}}}_{s}[\hat{Q}],$$

where for the spontaneous emission,

10 $${{\mathscr{S}}}_{s}[\hat{Q}]=-\,\sum _{\mu ,\nu =1}^{N}\frac{{F}_{\mu \nu }}{2}\,({\hat{\sigma }}_{\mu }^{+}{\hat{\sigma }}_{\nu }^{-}\hat{Q}+\hat{Q}{\hat{\sigma }}_{\mu }^{+}{\hat{\sigma }}_{\nu }^{-}-2{\hat{\sigma }}_{\mu }^{+}\hat{Q}{\hat{\sigma }}_{\nu }^{-}).$$

The dipole operator is ${\hat{\sigma }}_{\mu }^{-}$ (${\hat{\sigma }}_{\mu }^{+}$) where ${\hat{\sigma }}_{\mu }^{-}$ $\equiv$ $|g{\rangle }_{\mu }\langle e|$ and ${\hat{\sigma }}_{\mu }^{-}$ $\equiv$ ${({\hat{\sigma }}_{\mu }^{+})}^{\dagger }$. The pairwise frequency shift *G*_*μν*_ and decay rate *F*_*μν*_ are^24^

11 $${F}_{\mu \nu }(\xi )\equiv \frac{3{\rm{\Gamma }}}{2}\{[1-{(\hat{{\bf{p}}}\cdot {\hat{r}}_{\mu \nu })}^{2}]\frac{\sin \,\xi }{\xi }+[1-\mathrm{3(}\hat{{\bf{p}}}\cdot {\hat{r}}_{\mu \nu }{)}^{2}](\frac{\cos \,\xi }{{\xi }^{2}}-\frac{\sin \,\xi }{{\xi }^{3}})\},$$

12 $${G}_{\mu \nu }(\xi )\equiv \frac{3{\rm{\Gamma }}}{4}\{-\mathrm{[1}-{(\hat{{\bf{p}}}\cdot {\hat{r}}_{\mu \nu })}^{2}]\frac{\cos \,\xi }{\xi }+\mathrm{[1}-3{(\hat{{\bf{p}}}\cdot {\hat{r}}_{\mu \nu })}^{2}](\frac{\sin \xi }{{\xi }^{2}}+\frac{\cos \xi }{{\xi }^{3}})\},$$

where Γ is the single-particle natural decay rate of the excited state, *ξ* = |**k**_*L*_|*r*_*μν*_, and the interparticle distance *r*_*μν*_ = |**r**_*μ*_ − **r**_*ν*_|. The above expressions are valid for uniformly polarized excitations of the dipole orientations **p**.

The time evolutions of the HPI states can be determined by the eigenvalues and eigenvectors of the coupling matrix $\hat{M}$ with ${M}_{\mu \nu }=(\,-\,{F}_{\mu \nu }+i2{G}_{\mu \nu }{\delta }_{\mu \ne \nu }){e}^{-i{{\bf{k}}}_{L}\cdot ({{\bf{r}}}_{\mu }-{{\bf{r}}}_{\nu })}\mathrm{/2}$ in the bare state bases |*ψ*_*μ*_〉. Denote the eigenvalues and eigenvectors as *λ*_*m*_ and $\hat{U}$ respectively, the time evolution of the HPI state |Ψ(*t*)〉 = *h*_*l*_(*t*)|Φ_*l*_〉_HPI_ reads^37,38,41^

13 $${h}_{l}(t)=\sum _{m=1}^{N}\,{v}_{m}(l){e}^{{\lambda }_{m}t}{w}_{m}(l),$$

14 $${v}_{m}(l)=\sum _{{\mu }_{z}=1}^{{N}_{z}}\,\sum _{{\mu }_{\varphi }=1}^{{N}_{\varphi }}\frac{{U}_{\mu m}}{\sqrt{N}}{e}^{-i\frac{2l\pi }{{N}_{\varphi }}({\mu }_{\varphi }-\mathrm{1)}},$$

15 $${w}_{m}(l)=\sum _{{\nu }_{z}=1}^{{N}_{z}}\,\sum _{{\nu }_{\varphi }=1}^{{N}_{\varphi }}\frac{{U}_{m\nu }^{-1}}{\sqrt{N}}{e}^{i\frac{2l\pi }{{N}_{\varphi }}({\nu }_{\varphi }-\mathrm{1)}},$$

where the atomic position index *ν* is implicitly (*ν*_*z*_ − 1)*N*_*ϕ*_ + *ν*_*ϕ*_, which is the same as *μ*. The eigen-spectrum of *λ*_*m*_ involves both super- and subradiant decay rates along with the associated frequency shifts, and |*v*_*m*_(*l*)|^2^ is essentially the fidelity of |Φ_*l*_〉_HPI_ to the *m*th eigenstate, while |*v*_*m*_(*l*)*w*_*m*_(*l*)|^2^ gives a measure of how much *λ*_*m*_ contributes to the HPI state dynamics.

### Far-field scattering

The far-field scattering properties provide measurable information for characterizing the HPI states and the atomic system. Similar ring lattice has been used to prepare Rydberg states^76^ which show collective effects in the photon emissions. Here we use the general expression of the far-field scattering from the two-level atoms in Heisenberg picture^24^,

16 $$\langle {\overrightarrow{E}}^{\ast }({\bf{R}},t^{\prime} )\overrightarrow{E}({\bf{R}},t)\rangle ={(\frac{{\omega }_{eg}^{2}}{4\pi {\varepsilon }_{0}{c}^{2}})}^{2}\frac{|\overrightarrow{p}{|}^{2}}{{R}^{2}}[1-{(\hat{{\bf{R}}}\cdot \hat{{\bf{p}}})}^{2}]\sum _{\alpha ,\beta =1}^{N}{e}^{i{{\bf{k}}}_{R}\cdot {{\bf{r}}}_{\alpha \beta }}\langle {\hat{\sigma }}_{\alpha }^{+}(t^{\prime} ){\hat{\sigma }}_{\beta }^{-}(t)\rangle ,$$

where *ω*_*eg*_ is the energy difference, *R* = |**R**|, **r**_*αβ*_ = **r**_*α*_ − **r**_*β*_, and the orientation of the dipole moment $\overrightarrow{p}$ is determined by the polarization of the excitation. The far-field derivation assumes that the observation point is far compared to the size of the atomic ring, such that ${\omega }_{eg}|{\bf{R}}-{{\bf{r}}}_{\alpha }|/c\gg 1$. This also suggests that the radiation mode **k** ≈ **k**_*R*_//**R**_*α*_ [=(**R** − **r**_*α*_)] in Fig. 1 in the main paper, which indicates of the retarded phase ${e}^{i{k}_{R}({R}_{\alpha }-R)}\approx {e}^{-i{{\bf{k}}}_{R}\cdot {{\bf{r}}}_{\alpha }}$. Similar and more general expression can also be derived for a four-level atomic system^77^ (three Zeeman levels in the *J* = 1 excited state, as in strontium atoms), which takes equation (16) in a tensor form of dipole transitions.

Set the time *t* = *t*′ in equation (16), we can calculate the radiation field intensity in terms of the dipole operators in Schrödinger picture, that is, $\langle {\rm{\Psi }}(t)|{\hat{\sigma }}_{\alpha }^{+}{\hat{\sigma }}_{\beta }^{-}|{\rm{\Psi }}(t)\rangle$. Therefore, by substituting the HPI states |Ψ(*t*)〉 = *h*_*l*_(*t*)|Φ_*l*_〉_HPI_, we obtain the radiation field intensity,

17 $$\frac{{\langle {\overrightarrow{E}}^{\ast }({\bf{R}},t)\overrightarrow{E}({\bf{R}},t)\rangle }_{l}}{{I}_{0}(t)}=[1-{(\hat{{\bf{R}}}\cdot \hat{{\bf{p}}})}^{2}]\sum _{\alpha ,\beta =1}^{N}{e}^{i({{\bf{k}}}_{R}-{{\bf{k}}}_{L})\cdot {{\bf{r}}}_{\alpha \beta }}\frac{1}{N}\,{e}^{\frac{i2l\pi }{{N}_{\varphi }}({\beta }_{\varphi }-{\alpha }_{\varphi })},$$

where *I*_0_(*t*) = ${I}_{n}{h}_{l}^{\ast }(t){h}_{l}(t)$ is the time-evolving fluorescence intensity with ${I}_{n}\equiv {({\omega }_{eg}|\overrightarrow{p}|)}^{2}\mathrm{/(4}\pi {\varepsilon }_{0}{c}^{2}R{)}^{2}$, and again *α*(*β*) has an implicit dependence of *α*_*ϕ*_(*β*_*ϕ*_). Equation (17) characterizes the far-field scattering property from the HPI states prepared by an excitation field of *l*^th^ OAM, which involves the interplay of the atomic distributions **r**_*αβ*_ and the imprinted phases ${e}^{i2l\pi /{N}_{\varphi }}$.

### Mode symmetry

The mode symmetry of Ω_*l*_(*θ*, *ϕ*) = Ω_−*l*_(*θ*, *ϕ*) = Ω_*N*−*l*_(*θ*, *ϕ*) for even number of the atoms can be explained in a polygon geometry with the pairwise and spatial phase contributions of **k**_*R*_ · **r**_*αβ*_. These include a combination of ${C}_{2}^{N}$ cosine functions with *N* nearest-neighbor components (*β* = *α* + 1) and with *N*(*N* − 3)/2 diagonals in the geometry of *N*-polygon. The nearest-neighbor components pair up and interchange with *l* and −*l*. For the diagonals, they can be further grouped into (*N*/2 − 2) different lengths (next nearest-neighbor, next next nearest-neighbor, etc.) with *N* components respectively, and the diagonal with the maximal length (2*r*) with *N*/2 components. Again the *N* components in the respective groups can be interchanged with *l* and −*l*. The *N*/2 components in the maximal diagonal go back to themselves as *l* → −*l*. This is due to the form of $\cos (l\pi +{{\bf{k}}}_{R}\cdot {{\bf{r}}}_{\alpha \beta })$ which is the same as $\cos (\,-\,l\pi +{{\bf{k}}}_{R}\cdot {{\bf{r}}}_{\alpha \beta })$ with a phase difference of 2*lπ*.
